# Identification, quantification and age-related changes of human trabecular meshwork stem cells

**DOI:** 10.1186/s40662-019-0156-z

**Published:** 2019-10-17

**Authors:** Yogapriya Sundaresan, Muthukkaruppan Veerappan, Krishnadas Subbiah Ramasamy, Gowri Priya Chidambaranathan

**Affiliations:** 1 Department of Immunology and Stem Cell Biology, Aravind Medical Research Foundation, Madurai, Tamil Nadu India; 2 Glaucoma Clinic, Aravind Eye Hospital and Post Graduate Institute of Ophthalmology, Madurai, Tamil Nadu India

**Keywords:** Human trabecular meshwork, Trabecular meshwork stem cells, Two-parameter analysis, Age-related changes, Primary open angle glaucoma

## Abstract

**Background:**

Loss of cells in the human trabecular meshwork (TM) has been reported with ageing and in glaucoma. This study aims to identify, quantify and determine the age-related changes of human TM stem cells (TMSCs).

**Methods:**

Isolation of TM cells/ paraffin sectioning was carried out using human corneoscleral rings and whole globes. The TM cells/ sections were immunostained for the stem cell markers ATP-binding cassette protein G2 (ABCG2), nerve growth factor receptor p75 and AnkyrinG (AnkG). Images were acquired using Leica SP8 confocal microscope. The isolated cells were analyzed for two parameters- ABCG2 expression and nucleus to cytoplasmic ratio (N/C ratio). The total number of TM cells and those positive for ABCG2 and p75 in each section were quantified. Spearman rank order correlation was used to determine the association between age and the cell counts.

**Results:**

The TMSCs were identified based on two parameters- high ABCG2 expression and high N/C ratio > 0.7. These stem cells were also positive for p75 and AnkG. The TMSC content based on the two parameters was 21.0 ± 1.4% in < 30 years age group, 12.6 ± 6.6% in 30–60 years and 4.0 ± 3.5% in > 60 years. The stem cells with high ABCG2 and p75 expression were restricted to the Schwalbe’s line region of the TM. A significant correlation was observed between the reduction in TMSC content and TM cell count during ageing.

**Conclusion:**

The human TMSCs were identified and quantified based on two parameter analysis. This study established a significant association between age-related reduction in TMSC content and TM cell loss.

## Background

The human trabecular meshwork (TM) is a tiny porous tissue located at the iridocorneal angle of the eye, which serves as the conventional outflow pathway for the drainage of aqueous humor (AH). TM is neural crest in origin [1] and is organized to act as a mechanical pump that regulates the AH outflow to maintain intraocular pressure (IOP). Earlier studies have reported a loss of 6000 TM cells per year with ageing [2, 3]. In addition to TM cell loss, trabecular thickening, fusion of the trabeculae, extracellular matrix modification and loss of giant vacuoles from Schlemm’s canal endothelium [3, 4] contribute to increased IOP. The increase in IOP due to pronounced loss of TM cells has been reported in primary open angle glaucoma (POAG) [5]. Elevated IOP affects the optic nerve through the mechanical changes at the lamina cribrosa leading to irreversible blindness [6].

The TM comprises of two anatomical regions: (i) the filtering meshwork which facilitates the AH outflow and (ii) the anterior non-filtering region that is inserted beneath the peripheral corneal endothelium. Earlier reports have described the presence of unusually prominent clusters of the epithelioid cells with high nucleus to cytoplasmic (N/C) ratio at the junction of the anterior non-filtering region of TM in *Macaca mulata* [7] referred as the Schwalbe’s line cells. The presence of stem-like cells in this region was evident from active cell proliferation after argon laser trabeculoplasty in corneoscleral explant organ culture [8]. Recent studies on primate and bovine eyes have reported the presence of stem/ progenitor cells which are characterized by long term BrdU retention and OCT4 immunoreactivity in the Schwalbe’s line region/ transition zone [9, 10]. These putative stem cells have been shown to give rise to both corneal endothelium and trabeculae when required [10, 11]. However, specific markers for stem cells in human TM have not been identified yet. Characterization of cultured trabecular meshwork stem cells (TMSCs) expressed putative stem cells markers such as ATP-Binding Cassette G2 protein (ABCG2), NOTCH-1, MUC1 and AnkyrinG (AnkG). These cells were multipotent, had the ability to differentiate into TM cells with phagocytic property and home to TM when injected into the anterior chamber [12, 13]. Transplantation of iPSC-derived TM cells activated endogenous TM cell proliferation to repopulate the TM, thus reducing the IOP [14–16]. However, the role of TMSCs in maintaining tissue homeostasis and its fate in ageing remains unexplored. We hypothesize that TMSCs play an important role in maintaining tissue homeostasis and are reduced upon ageing compromising the tissue function.

Therefore, the current study is focused on identifying and quantifying the putative stem cells in the human TM in isolated native TM cells using ABCG2, a universal stem cell marker [17], nerve growth factor receptor p75, a neural crest derived stem cell marker [18] and AnkG, a stem cell marker [12] specifically expressed in the transition zone/ Schwalbe’s line region [10]. A combination of two parameters- high ABCG2 expression and high N/C ratio was used to identify and quantify TMSCs which was previously established to be a specific method for identifying human limbal epithelial stem cells [19]. Further, the location of TMSCs was determined in human tissue sections using the same stem cell markers and the cells expressing these markers were quantified. This study also elucidated the changes in the TMSC content with ageing and its correlation with total TM cell loss.

## Methods

### Sample collection

The whole globes not suitable for corneal transplantation from donors of age group < 30 years (younger age group), 30–60 years (middle age group) and > 60 years (older age group) (*n* = 3 each) were obtained from Rotary Aravind International Eye Bank, Madurai. The inclusion criteria for the selection of tissues were (i) eyes enucleated within 4 h of death and received within 24 h for research, (ii) donors with no history of ocular infection or systemic disease. Eyes from donors whose cause of death was due to poison or snake bite were excluded from the study. The corneoscleral rims of the three different age groups (*n* = 5 pairs each) obtained after corneal transplantation were used for the isolation of native TM cells. The study adhered to the tenets of the declaration of Helsinki and was approved by the Institutional Review Board of Aravind Eye Care System (IRB number: RES2016057BAS).

### Native TM cell isolation and cytosmear preparation

The TM was dissected from the corneoscleral rims under a dissection microscope (Nikon SMZ645-Japan). The TM was digested using collagenase A (4 mg/ml) (Roche- Basel, Switzerland) for 2 h at 37 °C. Following the digestion, the cells were centrifuged at 1200 rpm for 10 min at 4 °C (Heareus Primo Biofuge, Germany) [20]. Trypan blue assay was carried out to determine cell viability and cell count was determined with a hemocytometer (Sigma Aldrich, St. Louis, Missouri). Cytosmears of 2.5 × 10^4^ cells per slide were prepared by centrifuging at 400 rpm for 3 min using a cytospin system (Thermo Shandon – Pittsburg, PA). The TM cell cytosmears were fixed in ice cold acetone followed by immunostaining.

### Paraffin sectioning

The anterior segments of the eyes were dissected with intact iris/ ciliary body and divided into four quadrants. Following fixation in 10% buffered formalin for 24 h, quadrants were embedded in paraffin and sectioned (5 μm). The sections were deparaffinized and antigen retrieval was carried out using 10 mM citrate buffer (pH 6.4) for 20 min at 90 °C followed by immunostaining [21].

### Immunostaining

The sections and cytosmears were blocked with avidin biotin blocking system (DAKO- Glostrup, Denmark). Mouse monoclonal anti- BCRP antibody (anti-ATP- Binding Cassette G2-Millipore, Billerica, MA) was added at a dilution of 1:20 in 5% BSA in 1X PBS (Sigma Aldrich, St. Louis, Missouri). After overnight incubation at 22 °C, biotinylated secondary antibody (Goat anti- mouse IgG, DAKO-Glostrup, Denmark) was added at a dilution of 1:200 in 5% BSA and incubated for 1 h at 22 °C. Visualization was carried out using streptavidin- fluorescein isothiocyanate (FITC, BD Pharmingen- San Diego, CA) at a dilution of 1:1000 in 1X PBS for 1 h at 22 °C. For double immunostaining, rabbit anti-human p75 antibody (Promega- Madison, Wisconsin) / anti-AnkG antibody (Millipore, Billerica, MA) was added at a dilution of 1:100 in 5% BSA in 1X PBS. After overnight incubation, biotinylated secondary antibody (Mouse anti- rabbit IgG, Santa Cruz Biotechnology Inc- San Francisco, CA) was added at a dilution of 1:200 in 5% BSA and incubated for 1 h at 22 °C. Visualization of p75/ AnkG staining was carried out using streptavidin Alexa Fluor 633 (Thermofisher Scientific- Waltham, Massachusetts) at a dilution of 1:500 in 1X PBS. Between the steps, the slides were washed with 1X PBS. The stained sections and cytosmear were then mounted with Vectashield mounting medium (Burlingame, CA) containing DAPI/propidium iodide (PI). Cytospin smears/ paraffin sections without adding primary antibody during the immunostaining were used as negative control.

### Confocal microscopy and N/C ratio calculation

Acquisition of confocal images was carried out using a laser scanning microscope (Leica SP8 confocal microscope, Germany) as previously described [22]. Briefly, fluorescent Z stack images were acquired with the following settings: the emission band width for FITC ranged from 496 to 535 nm using laser blue 488; for PI from 550 to 600 nm using laser green 552 nm and for Alexa Fluor 633 from 610 to 725 nm using laser red 633 nm. Using the above parameters, the images were acquired from the Schwalbe’s line region till the posterior region of the meshwork where the TM attaches with ciliary body. The Z stack images of 100 consecutive TM cells were acquired from double immunostained cytosmear for FITC, Alexa Fluor 633, PI and bright field using 40X objective zoom 2.

### Two parameter analysis

From the Z stack images of TM cells, the cellular and nuclear areas were measured using Leica Software (LAS AF 3.3.0.10134). The N/C ratio of the TM cells was calculated in Microsoft Excel by dividing the nuclear and cytoplasmic area [22]. The fluorescence intensity was quantified based on mean pixel intensity after reconstructing the Z stack image to a 2D maximum projection along a fixed axis. The level of ABCG2 expression was quantified based on the mean pixel intensity of the membrane staining using three linear Region of Interest (ROI) of equal length [19]. The cells with mean pixel intensity 188 ± 24 (Mean ± SD) were graded high positive (++), 125 ± 42 positive (+) and 53 ± 28 as negative for ABCG2. Similarly, the expression of p75 and AnkG was graded either positive or negative. The TMSCs were identified based on two parameter analysis which was established as a specific method to identify and quantify human limbal epithelial stem cells [19]. A scatter plot was constructed with ABCG2 intensity as X-axis and N/C ratio as Y-axis. The plot was divided into four quadrants at X = 0.7 and Y > 1. The upper right cells with N/C ratio > 0.7 and ABCG2 positivity =2 were designated as putative stem cells of the TM.

### Quantification of total TM cell count and immunopositive cells in TM sections

**Fig. 1:**
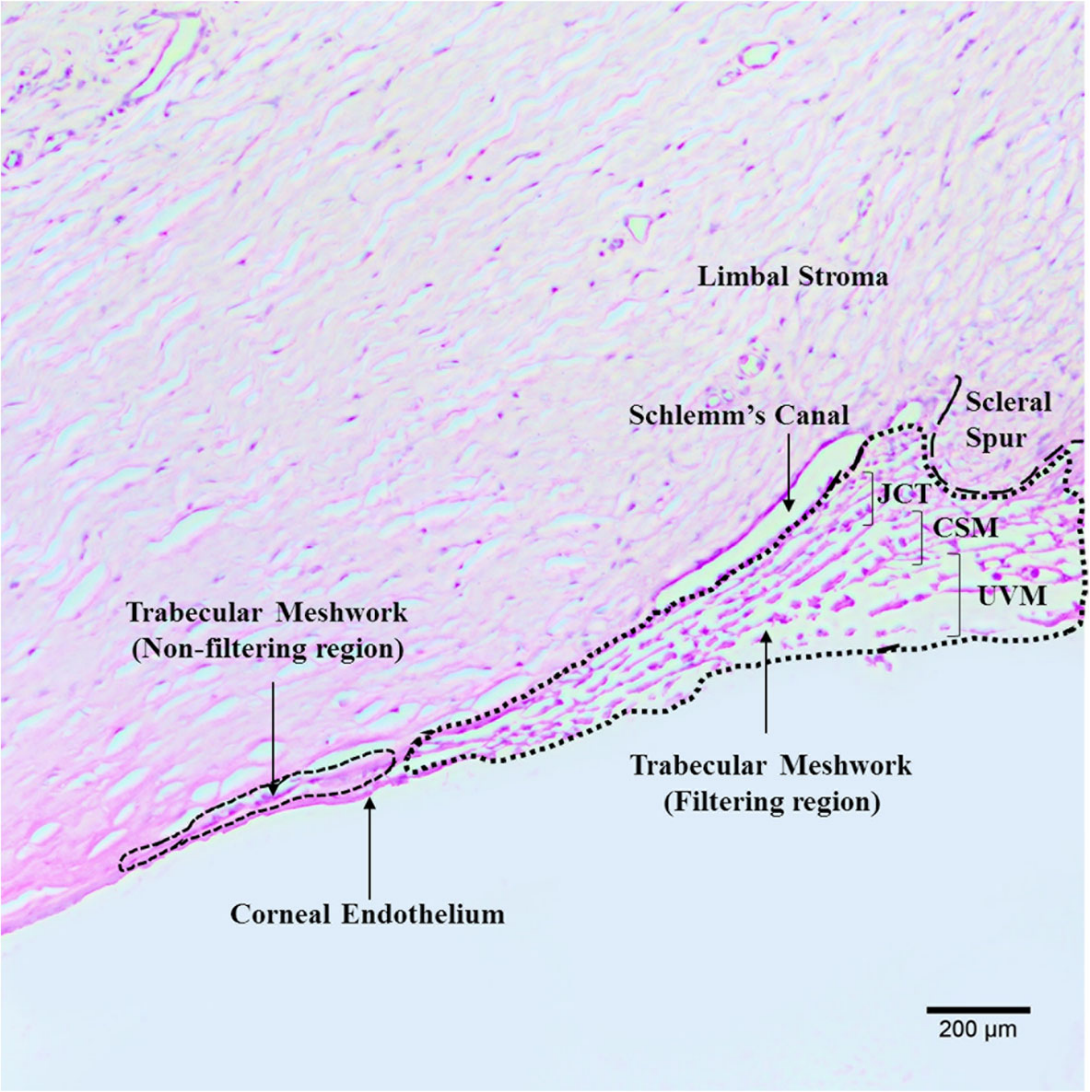
Hematoxylin and eosin stained human TM section to demarcate the filtering and non-filtering region

Two quadrants from each eye were included in the study. A minimum of three sections per quadrant were analyzed. The total number of cells in the TM [including the filtering region and the non-filtering region excluding the Schlemm’s canal endothelium (Fig. 1)] were quantified based on DAPI staining. Cells highly positive for ABCG2 and p75 in each section were also counted. The percentage of cells immunopositive for ABCG2 and p75 among the total number of nuclei in the TM were calculated. The mean total cell count and ABCG2/ p75 positive cells was averaged for each age group.

### Statistical analysis

Spearman Rank Order Correlation was performed to determine the statistical significance between the age and the cell counts using Stata 14.0 and a *p* value of less than 0.05 was considered statistically significant.

## Results

### Identification of human TMSCs in isolated TM cells by two parameter analysis

**Fig. 2:**
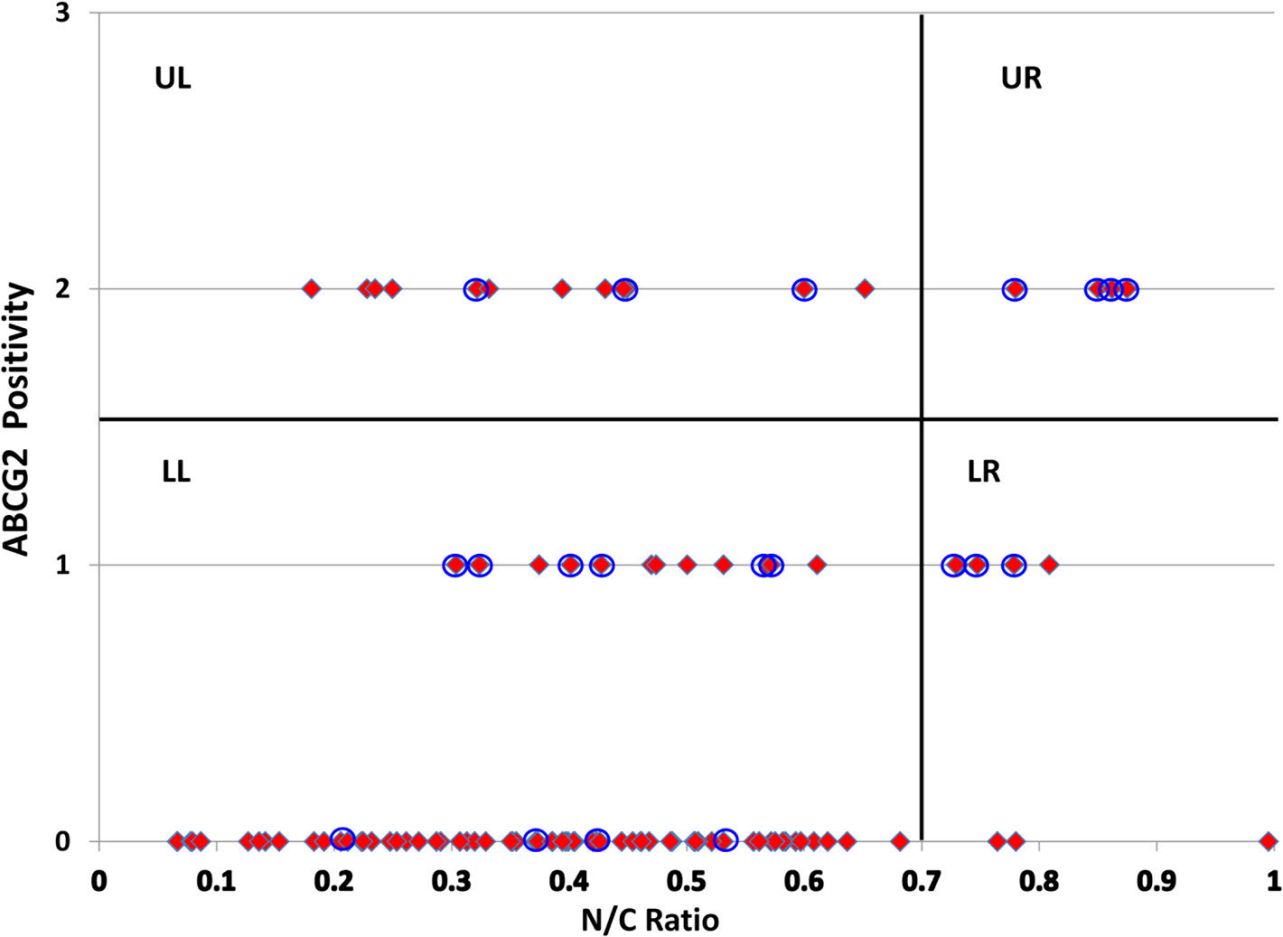
Representative scatter plot with two parameters (ABCG2 positivity versus N/C ratio) indicating that the stem cells in the upper right (UR) quadrant were strongly positive for ABCG2 and had high N/C ratio. UL: upper left, LL: lower left; LR: lower right. Each red diamond represents a cell. Dark blue circle denotes that the cell was p75 positive. Cells with no circle were negative for p75. All the cells in the UR quadrant were positive for p75

**Fig. 3:**
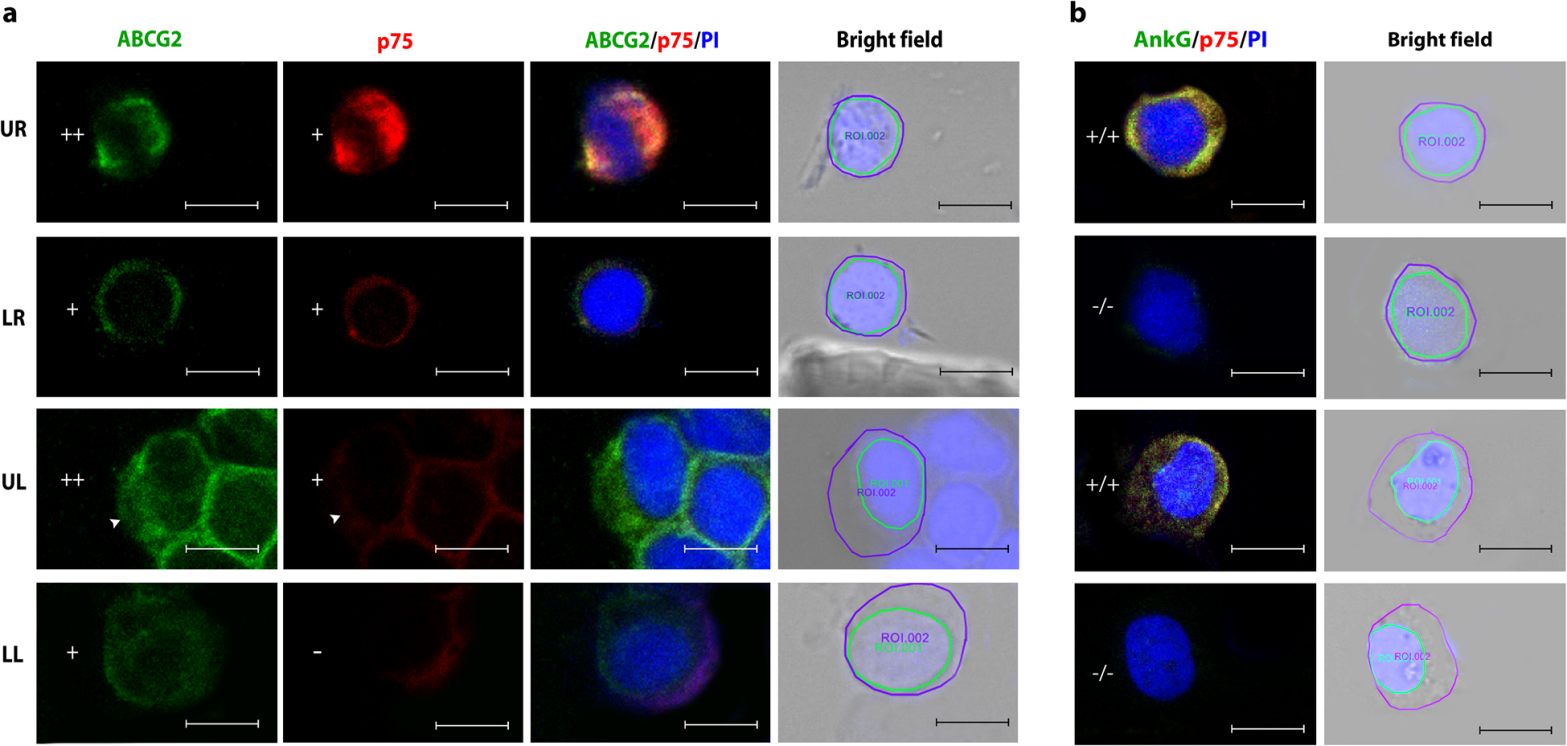
Representative confocal images of isolated TM cell cytosmears immunostained for (**a**) ABCG2 (FITC-green) and p75 (Alexa 633-red) and (**b**) AnkG (FITC-green) and p75 (Alexa 633-red), counterstained with propidium iodide (PI-blue). The cells with mean pixel intensity 188 ± 24 (Mean ± SD) were graded high positive (++), 125 ± 42 positive (+) and 53 ± 28 as negative for ABCG2. The expression of p75 and AnkG was graded either positive (+) or negative (−). Nuclear and cytoplasmic area were measured by marking the region of interest (ROI) around each nucleus and cell on the PI/bright field overlay image. The cells in the upper right (UR, first row) quadrant of the scatter plot were highly positive for ABCG2 with high N/C ratio and these were designated as stem cells. These UR cells also expressed p75 and AnkG. Lower right (LR, second row) quadrant cells had high N/C ratio but low ABCG2 expression. The white arrow head indicates a cell in the upper left (UL, third row) quadrant having high ABCG2 expression but low N/C ratio and lower left (LL, fourth row) quadrant cells had low ABCG2 expression and low N/C ratio. +/+ − cell positive for both AnkG and p75; −/− cell negative for AnkG and p75. Scale bar 10 μm

The TM cells were analyzed for two-parameters – level of ABCG2 expression and N/C ratio. Based on these parameters, a scatter plot was prepared (Fig. 2) and divided into four quadrants. The upper right (UR) quadrant cells were characterized by high ABCG2 expression and high N/C ratio, a feature of stem cells. The upper left (UL) quadrant cells expressed high levels of ABCG2 but had low N/C ratio. The lower left (LL) quadrant cells were characterized by minimal or no ABCG2 expression and low N/C ratio. Though the lower right (LR) quadrant cells had high N/C ratio, the expression of ABCG2 was either minimal or absent (Fig. 3).

Double immunostaining of the TM cytosmears for ABCG2 and p75 indicated that all the UR cells with high ABCG2 expression and high N/C ratio were also positive for the neural crest derived stem cell marker p75 (Fig. 3a). In parallel, double immunostaining for p75 and AnkG identified their co-expression in the UR cells (Fig. 3b). Thus, in addition to the two-parameters, p75 and AnkG expression confirms the stem cell property of the UR cells.

The above data also revealed that high expression of ABCG2, p75 and AnkG positivity was not restricted to the UR quadrant cells signifying the importance of combining another parameter such as N/C ratio to identify TMSCs.

### Location of human TMSCs

**Fig. 4:**
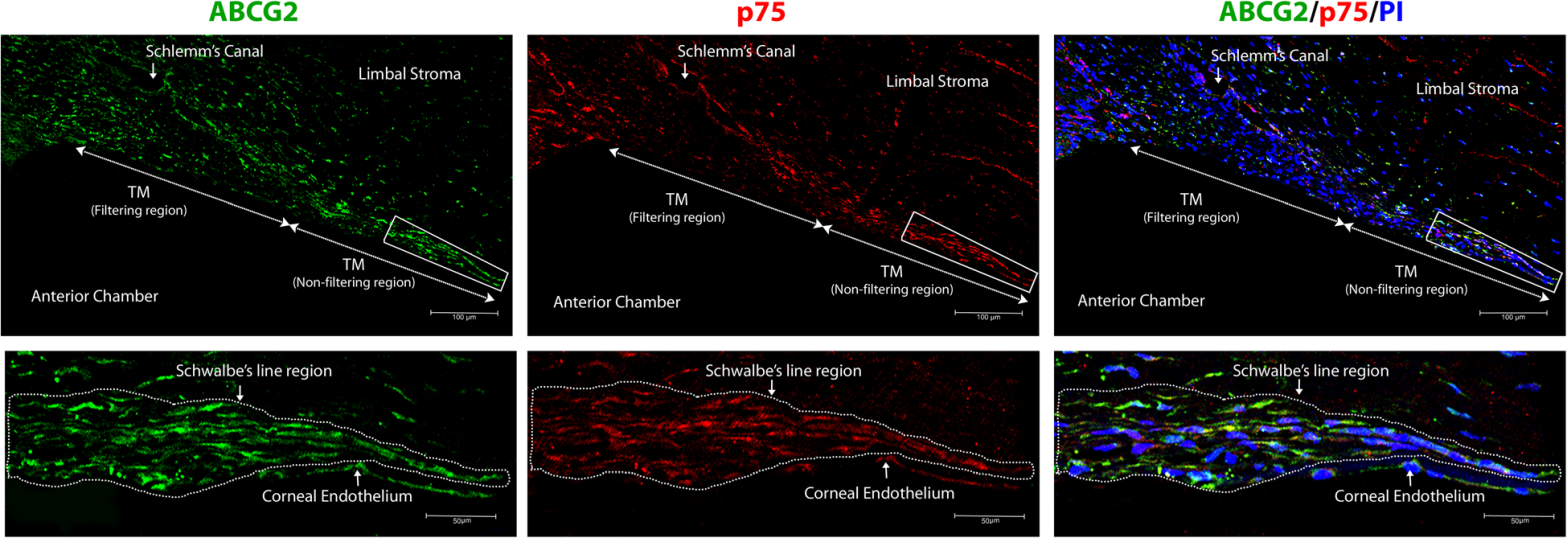
Representative confocal images of TM double immunostained for ABCG2 (FITC-green), p75 (Alexa 633- red) and overlay of ABCG2 and p75 expression with nuclear counterstain PI (blue). The magnified images of the Schwalbe’s line region (dotted region in the lower magnification) is shown below. Immunostaining of TM sections identified ABCG2 high positive and p75 positive cells to be restricted to the Schwalbe’s line region of the human TM

Immunostaining of the radial TM paraffin sections revealed the expression of ABCG2 throughout the meshwork (filtering and non-filtering region). High ABCG2 expression, a characteristic feature of stem cells, was observed in all the cells of the Schwalbe’s line region in the non-filtering meshwork whereas the cells in the filtering region had lower or minimal expression (Fig. 4).

Similar to the ABCG2 staining pattern, the immunostaining of neural crest derived stem cell marker p75, identified all the cells in the Schwalbe’s line region to be positive. In contrast, the cells in the filtering meshwork were negative for p75 (Fig. 4). Double immunostaining of the TM for ABCG2 and p75 (*n* = 3 donor tissues) indicated co-expression of high ABCG2 and p75 in the Schwalbe’s line region (Fig. 4).

### Age- related changes in TM

#### TMSC content in isolated TM cells

**Fig. 5:**
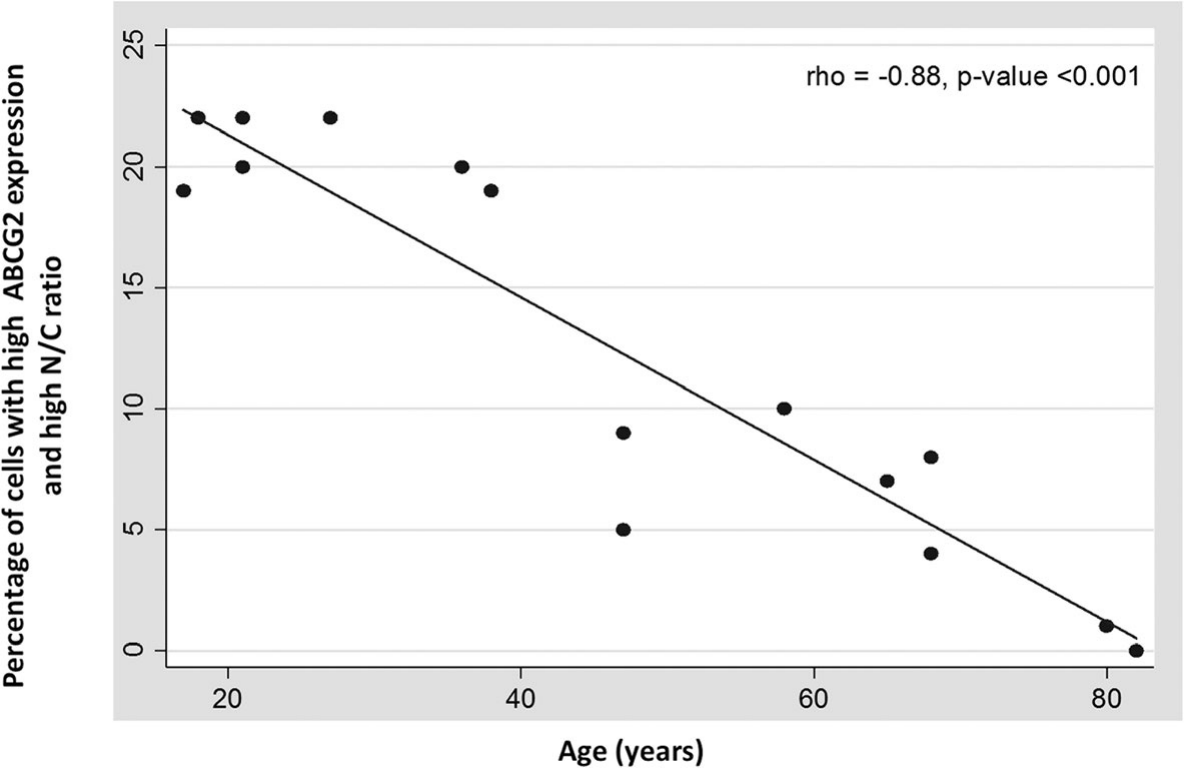
Correlation plot between age and stem cell content in isolated native TM cells (rho = − 0.88, *p* < 0.001) revealed a significant decrease in TMSC content with ageing. Each dot in the plot represents the percentage of cells with high ABCG2 expression and high N/C ratio in a single donor

Based on two-parameter analysis, the percentage (mean ± SD) of cells in each quadrant of the scatter plot per age group is represented in Table 1. The percentage of stem cells with high ABCG2 expression and high N/C ratio (UR cells) in younger donors (< 30 years) was observed to be 21.0 ± 1.4%. This percentage decreased significantly to 12.6 ± 6.6% in middle (30–60 years) and 4.0 ± 3.5% (> 60 years) in older age group (rho = − 0.88 and *p* < 0.001). The Spearman rank correlation plot indicated a negative correlation between age and stem cell count in isolated native TM cells (Fig. 5).

**Table 1:** Distribution of cells in the four quadrants of the scatter plot in three different age groups. The UR cells with high ABCG2 expression and high N/C ratio were designated as stem cells. The stem cell content significantly decreased with ageing (rho = − 0.88 and *p* < 0.001)

Age group	Percentage of cells (Mean ± SD)			
	LL	UL	LR	UR
< 30 years	29.75 ± 10.2	43.0 ± 12.4	5.75 ± 4.6	21.0 ± 1.4
30–60 years	37.0 ± 20.5	44.2 ± 19.8	6.8 ± 5.8	12.6 ± 6.6
> 60 years	65.0 ± 18.2	23.8 ± 13.6	7.4 ± 4.7	4.0 ± 3.5

*UR* = upper right; *UL* = upper left; *LL* = lower left; *LR* = lower right

#### TM sections

##### Quantification of total TM cellularity

**Fig. 6:**
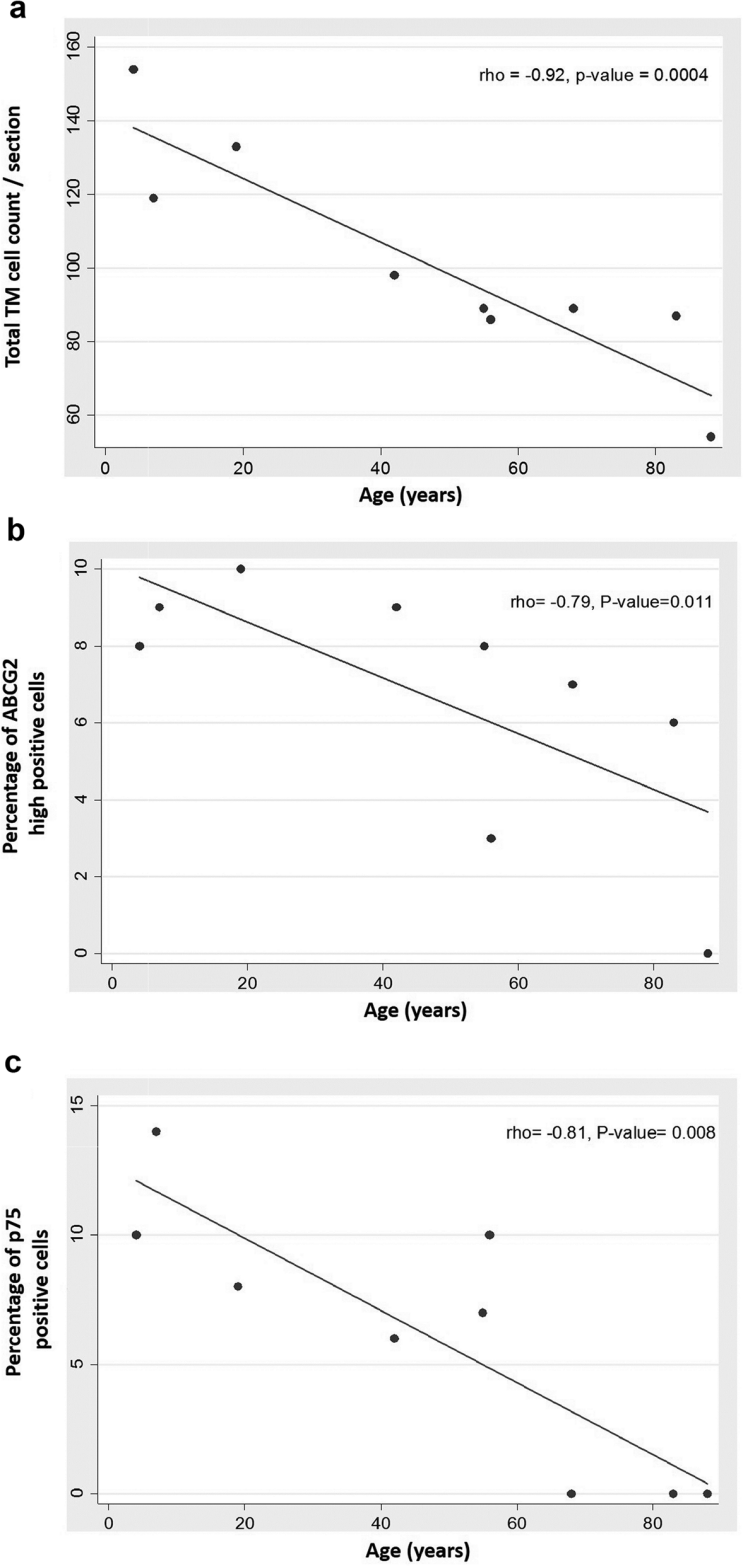
Correlation plot between age and (**a**) total TM cell count (rho = − 0.92, *p* = 0.0004), (**b**) percentage of ABCG2 high positive cells (rho = − 0.79, *p* = 0.011) and (**c**) percentage of p75 positive cells (rho = − 0.81 and *p* = 0.008). A significant decrease in total TM cell count and TM stem cell content was observed upon ageing. Each dot in the plot represents data from a single donor

Analysis of total TM cell count (mean ± SD) based on DAPI staining revealed the presence of 134 ± 30 cells per section in younger age group, 93 ± 16 cells in middle age group and 80 ± 17 cells in donors from older age group. Spearman rank correlation analysis indicated a significant decrease in cell count in the middle age group when compared to the younger age group and this reduction was higher in older age group (rho = − 0.92; *p* = 0.0004) (Fig. 6a and Table 2).

**Table 2:** Age related changes in the TM. Total TM cell count, percentage of ABCG2 high and p75 positive cells decreased with ageing

Age group (*n* = 3 each)	Cell count/ section (Mean ± SD)		
	Total TM cell count	ABCG2 high positive (++) cells (%)	p75 positive (+) cells (%)
< 30 years	134 ± 30	9.4 ± 3.0	11.2 ± 4.4
30–60 years	93 ± 16	7.2 ± 4.0	7.1 ± 6.0
> 60 years	80 ± 17	5.4 ± 4.0	0.3 ± 1.0

##### Quantification of TMSC content

**Fig. 7:**
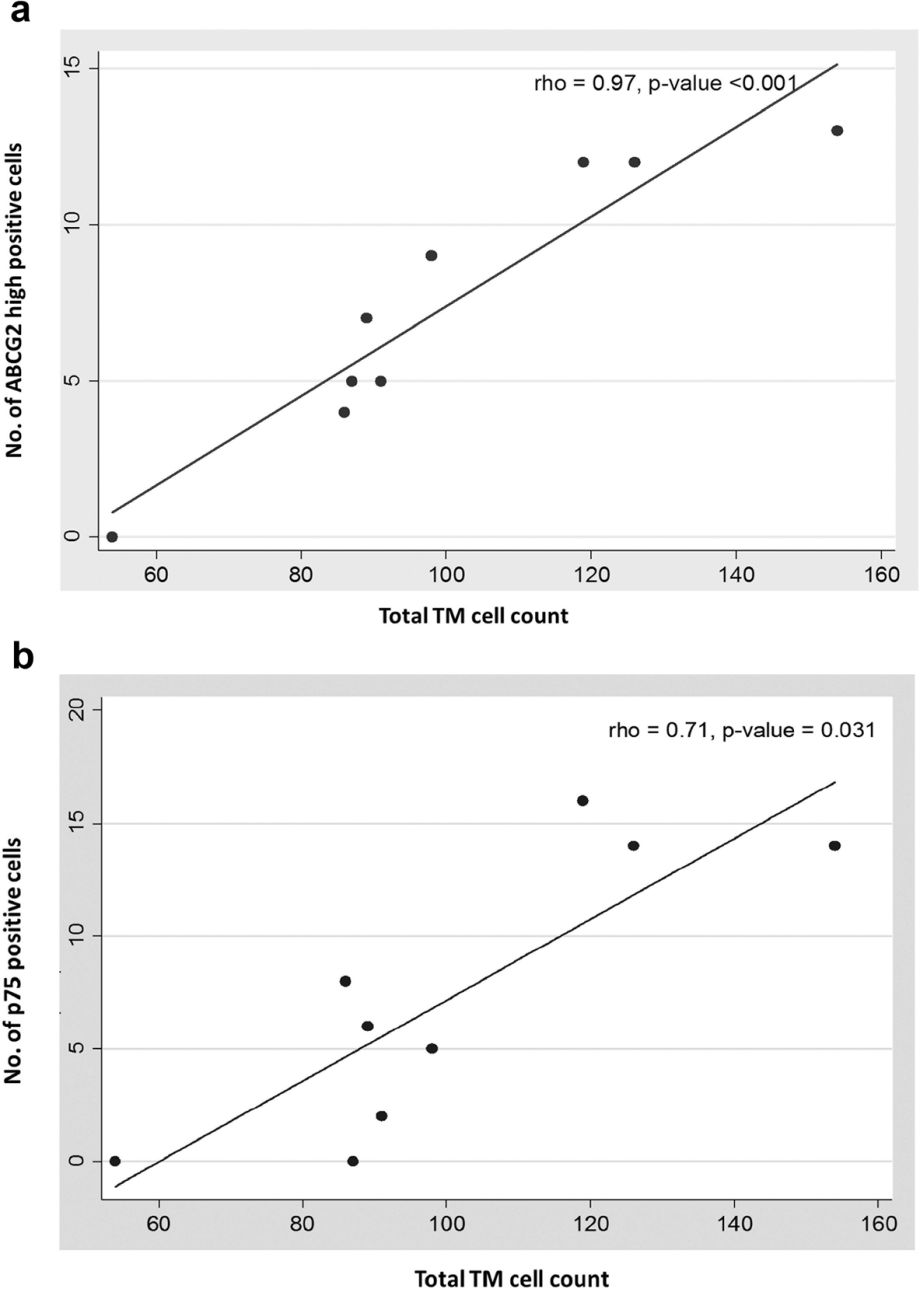
Correlation plot between total TM cell count and (**a**) ABCG2 high positive cells (rho = 0.97, *p*-value < 0.001) and (**b**) p75 positive cells (rho = 0.71, *p*-value =0.031). A significant decrease in total TM stem cell count was observed with the reduction in total TM cell count. Each dot in the plot represents data from a single donor

Analysis of confocal microscopic images of immunostained TM sections using ImageJ revealed that in younger donors, 9.4 ± 3.0% (mean ± SD) and 11.2 ± 4.4% of TM cells/ section had high positivity to ABCG2 and p75, respectively. This percentage of ABCG2 high positive and p75 positive cells significantly reduced to 7.2 ± 4.0% and 7.1 ± 6.0% in the middle age group and to 5.4 ± 4.0% and 0.3 ± 1.0% in the older age group [ABCG2 (rho = − 0.79; *p* = 0.011) (Fig. 6b); p75 (rho = − 0.81; *p* = 0.008) (Fig. 6c); (Table 2)]. Though there was a significant reduction in the percentage of both ABCG2 high positive and p75 positive cells, the loss in the percentage of p75 positive cells was observed to be higher in the older age group when compared to the loss of ABCG2 high positive cells. The Spearman rank correlation analysis between total TM cell count and ABCG2 high positive (rho = 0.97; *p* = < 0.001) (Fig. 7a) and p75 positive cells (rho = 0.71; *p* = 0.031) (Fig. 7b) indicated a significant association between loss in stem cell content with total TM cell reduction.

## Discussion

The current focus of research in glaucoma is to develop a cell-based therapy to regenerate the TM, thereby enhancing AH outflow facility. Reports on locating and characterizing TMSCs, as well as regenerating TM using TMSCs/iPSCs are available on animal models, primate and bovine eyes [7–10, 12–16]. However, there are only a few reports on human TM and none on the role of these adult tissue resident stem cells in maintaining TM homeostasis. Hence, this study focused on identifying and quantifying putative human TMSCs as well as determining the age-related changes in the TMSC content using native TM tissues and isolated cells.

Aside from one report that quantified TMSCs based on the expression of putative stem cell markers including ABCG2 and AnkG on bovine eyes [10], there is no specific method available to date that allows for the quantification of human TMSCs. Since there are no specific markers for adult stem cells, we previously established a two parameter analysis – by combining high expression of either p63 or ABCG2 with high N/C ratio as a specific method for the identification and quantification of both human limbal and buccal mucosal epithelial stem cells [19, 22, 23]. On the basis of this two- parameter analysis (high ABCG2 expression and high N/C ratio), the percentage of stem cells (UR cells) in the native human TM cells were identified in this study (Figs. 2 & 3). Based on the previous experience with human limbal epithelial stem cells, we hypothesize that the cells in the UL quadrant of the scatter plot to represent the transient amplifying cells as these cells expressed higher ABCG2 levels but had low N/C ratio. The LL cells in the scatter plot might represent the differentiated cell population with minimal or no ABCG2 expression and low N/C ratio. Though LR cells had high N/C ratio, these cells might represent the senescent cells due to the minimal expression or absence of ABCG2 expression (Figs. 2 & 3). Thus, either by the lower ABCG2 expression or low N/C ratio property, these cells were not considered stem cells. Further confirmation is essential using a differentiated cell marker which will be expressed exclusively by non-stem cell population. Endothelial cell contamination during TM cell isolation was ruled out by CD31 immunostaining (data not shown). The expression of neural crest derived stem cell marker p75 and putative TMSC marker AnkG provided additional proof to the fact that the UR quadrant cells represented TMSCs. Quantification of TMSCs demonstrated a significant reduction with ageing. Functional studies such as label retaining cell property (LRC) are essential to further confirm this method of identification of stem cells.

To determine the location of these TMSCs, the native human TM tissues were immunostained for the stem cell markers ABCG2 and p75. The expression of ABCG2 was observed throughout the TM as previously reported [10]. But a higher expression of ABCG2 was identified in the Schwalbe’s line region in this study. The expression of p75 was also restricted to this region. Raviola had earlier demonstrated that the cells in the Schwalbe’s line region to have high N/C ratio. Further, double immunostaining the TM sections (*n* = 3 donor eyes) for ABCG2 and p75 (Fig. 4) confirmed the location of the human TMSCs in the Schwalbe’s line region.

A significant reduction in TM cellularity was earlier established with ageing [2, 3]. To determine the status of TMSCs with ageing, the percentage of ABCG2 high and p75 positive cells in native human TM tissues were quantified in three different age groups. A significant decline in TMSCs expressing high ABCG2 and p75 in the process of ageing was evident in the current study. This reduction in stem cell content significantly correlated with the loss of TM cellularity with ageing. In support of our data, p75 expressing mouse adipose-derived stem cells revealed a significant reduction in the number with ageing [24]. In addition, side population cells from rat dental pulp tissues expressing high levels of ABCG2 declined with ageing [25]. However, certain adult stem cells such as hematopoietic stem cells and hair follicle stem cells do not decline quantitatively with age, but a clear loss of function was reported [26, 27].

The anatomical changes in the TM with the process of ageing includes accumulation of ECM in the meshwork, trabecular thickening, fusion of trabeculae and loss of giant vacuoles in the Schlemm’s canal endothelium [3, 4]. These factors might also affect the stem cells in the TM that express ABCG2 and p75 leading to the decline in number with ageing. However, further studies are essential to confirm the effect of these factors on the loss of stem cells. In addition, further studies are required to understand whether the loss of stem cells with ageing actually causes reduced TM cellularity.

## Conclusion

In conclusion, the current study has established the two-parameter analysis, high N/C ratio and high expression of ABCG2, as a method for identifying and quantifying putative human TMSCs. These TMSCs expressing higher levels of ABCG2 and p75 were restricted to the Schwalbe’s line region of the anterior non-filtering meshwork. In addition, quantification of the TMSCs revealed a strong correlation between age-related TM cell reduction and stem cell loss. It is further essential to understand the nature of these stem cells in glaucomatous condition wherein the TM cell loss is more pronounced. However, the role of these TMSCs in maintaining the tissue homeostasis remains unclear. This understanding on the basic biology of TMSCs would help in developing a better stem cell-based therapy for POAG patients.

## Data Availability

All data generated or analyzed during this study are included in this published article.

## Abbreviations

ABCG2: ATP-Binding Cassette G2 protein
AnkG: AnkyrinG
LL: Lower Left
LR: Lower Right
N/C ratio: Nucleus to Cytoplasmic ratio
TM: Trabecular Meshwork
TMSCs: Trabecular Meshwork Stem Cells
UL: Upper Left
UR: Upper Right
